# Effect of sex on histomorphometric properties of Langerhans islets in native chickens

**Published:** 2015-12-15

**Authors:** Ali Parchami, Sanaz Kusha

**Affiliations:** 1 *Department of Anatomical Sciences, Faculty of Veterinary Medicine, Shahrekord University, Shahrekord, Iran; *; 2 *Undergraduate student of Veterinary Medicine, Shahrekord University, Shahrekord, Iran. *

**Keywords:** Lobe fraction, Native chickens, Pancreatic islets, Sex

## Abstract

The aim of the present study was to investigate the effect of gender on the distribution of pancreatic islets in native chicken. Ten adult male and ten adult female Isfahan native chickens were used in this experiment. Results showed a distinct sexual dimorphism in the native chicken pancreas which depends upon the various fractions of the pancreatic lobes, which were occupied by alpha, beta and mixed islets. In both sexes, the islets were more frequently found in the splenic and the third lobes, whereas they were more scarcely observed in the ventral and the dorsal lobes. In both sexes, there were no alpha islets in the dorsal and ventral pancreatic lobes. The mean percentage of beta islets in the third and splenic lobes were significantly greater in males than females (*p *< 0.05). However, the mean percentage of mixed islets in the third and splenic lobes were significantly greater in females than males (*p* < 0.05). The mean percentage of the alpha islets in the splenic and third lobes and the mean percentage of beta and mixed islets in the dorsal and ventral lobes was similar in both sexes in chickens. There was no sex difference in the mean percentage of whole gland islets (*p* > 0.05).

## Introduction

Functionally, the pancreas is divided into two types, the exocrine, enzyme-secreting cells and the endocrine, hormone-producing cells.^1^ The endocrine portion of the birds pancreas occupies considerably more tissue mass than it does in mammals, and also the distribution of cell types differs considerably. A-, B-, D-, and F- pancreatic polypeptide (PP), cells comprise the avian endocrine islet, but the distribution within the islet as well as within different pancreatic lobes appears more random than almost logical distribution found in discrete mammalian pancreas. A-cells synthesize and release glucagon, B-cells synthesize and release insulin, D-cells somatostatin, and F-cells pancreatic polypeptide.^2^ Existence of various hormone producing cells was demonstrated in the pancreas of avian species including chicken,^3^^,^^4^ duck^5^ and mallard.^6^ However, no reports has been shown gender effects on the distribution of endocrine cell types in the pancreas of avian species. Keeping in mind that the location of pancreatic islets in each pancreatic lobe is a critical factor in experiments involving partial pancreatectomy, the aim of the present study was to determine gender effects on the morphological and histochemical properties of the endocrine pancreas using light microscopic examination in native chickens. 

## Materials and Methods

The structure of the pancreas was investigated in this study in 20 healthy adult Isfahan native chickens (10 male, 10 female) using histochemistry and light microscopy. Following euthanasia with an intravenous overdose of sodium thiopental (40 mg kg^-1^; Mercury Medicare, Chennai, India) the birds were submitted to arterial perfusion through the left cardiac ventricle with neutral formalin solution in 0.1 M phosphate buffer, pH 7.2. Tissue samples taken from the caput, corpus and cauda of each lobe were fixed in Bouin’s fixative fluid for 18 hr. The tissue samples were passed through a standard alcohol dehydration-xylene sequence and embedded in paraffin, and 5 μm thick serial sections cut from tissue blocks. The sections were stained by the Crossmon’s modified triple stain^7^ for the examination of the general histological structure of the organ and with the aldehyde fuchsin stain for demonstration of alpha, beta and mixed islets.^8^ The localization of the different islet types in the whole pancreas was semi-quantitatively determined from five serial sections. Each islet type was counted in randomly 10 microscopic fields for each serial section (using a 10× objective), then the arithmetic mean was calculated. Mean ± standard deviation values were calculated for each islet to determine the significance of inter-lobes differences. Each islet was analyzed separately using one-way analysis of variance (ANOVA). The Duncan test was used for determining differences among pancreatic lobes. The results of the repartition of the different islet types throughout the different pancreatic lobes were expressed as means ± standard deviations and were compared using the Student *t*-test in SPSS (version 16; SPSS Inc., Chicago, USA). Differences were considered as significant when *p*-values were less than 0.05. 

## Results

The native chicken pancreas which is located between the ascending and descending loops of the duodenum was determined to be composed of the dorsal, ventral, third and splenic lobes. The gland was containing round or irregular islets distributed throughout the pancreas that were comprised of alpha, beta and mixed islets. Results showed that in both sexes, all three types of pancreatic islets were observed in the pancreas with different amounts.

Quantitative analysis of alpha, beta and mixed islets according to the different lobes are shown in Table 1. The most important morphometric findings are as follow:

1. In both sexes, the endocrine pancreas was mainly composed of beta islets.
2. In both sexes, the endocrine islets were more frequently found in the splenic and the third lobes, whereas they were more scarcely observed in the ventral and the dorsal lobes.
3. In both sexes, there were no alpha islets in the dorsal or ventral pancreatic lobes.
4. Frequencies of beta islets in different lobes in male chickens were as follow: Splenic lobe > dorsal lobe > third lobe > ventral lobe and in females as: Splenic lobe > dorsal lobe > ventral lobe > third lobe.
5. In both sexes, frequencies of mixed islets in different lobes were as follow: Ventral lobe > dorsal lobe > third lobe > splenic lobe.
6. In both sexes, the mean proportion of alpha islets was significantly greater in third than in splenic lobe (*p* < 0.05).
7. The mean proportion of beta islets in the third and splenic lobes was significantly greater in male than in female chickens (*p* < 0.05).
8. The mean proportion of mixed islets in the third and splenic lobes was significantly greater in female than in male chickens (*p* < 0.05).
9. The mean proportion of the alpha islets in the splenic and third lobes and the mean proportion of beta and mixed islets in the dorsal and ventral lobes was similar in chickens of both sexes.
10. There was no difference in the mean proportion of the pancreatic islets in the whole gland in terms of sex.

**Table 1:** Proportions (%) of different islet types of the endocrine pancreas according to the lobes in native chickens (n = 10). The data are expressed as mean ± standard deviation

**Pancreatic islets**	**sex**	**Dorsal lobe**	**Ventral lobe**	**Third lobe**	**Splenic lobe**	**All pancreas**
**Alpha islets **	Male	0.00	0.00	28.33 ± 1.23	22.31 ± 1.35	25.32 ± 3.33
	Female	0.00	0.00	27.36 ± 1.24	20.56 ± 1.29	23.96 ± 3.70
**Beta islets **	Male	68.08 ± 3.28	56.49 ± 3.20	60.34 ± 1.99	72.38 ± 1.95	64.32 ± 6.83
	Female	62.56 ± 2.69	54.60 ± 2.76	52.10 ± 1.60	66.40 ± 1.69	58.85 ± 6.29
**Mixed islets**	Male	32.56 ± 3.68	44.38 ± 2.24	12.20 ± 1.06	6.24 ± 0.57	23.84 ± 15.65
Female	38.37 ± 3.05	46.17 ± 2.88	21.47 ± 1.20	14.38 ± 1.03	30.09 ± 13.02

## Discussion

In the present study, we investigated for the first time gender effects on histomorphometric properties of pancreatic endocrine islets in native chickens using histo-chemistry. The results revealed a distinct sexual dimorpism in the native chicken pancreas which depends upon the various fractions of the pancreatic lobes occupied by alpha, beta and mixed islets in male than in female native chickens.

Results of the present study revealed that the native chicken pancreas, like in quails^9^ and geese,^10^ is located between the two arms of the duodenal loops and also has dorsal, ventral, third and splenic lobes, whereas it is three lobed structure in ducks,^5^ and falcons.^11^ The endocrine pancreas of avian species is composed of small and large islets that are named as alpha, beta and mixed islets.^12^ Alpha islets stained with silver are also known as dark islets, whereas beta islets stained with silver are also known as light islets.^1^ Results obtained from the present study showed that like in the other birds, three types of Langerhans islets (alpha, beta and mixed) were clearly identified in the endocrine pancreas of native chickens of both sexes. 

Results obtained from this study also showed that in both sexes, the endocrine pancreas was mainly composed of beta islets. This finding was in agreement with that of previous studies in chickens,^13^ quails^14^ and geese.^10^

Our results showed that in both sexes, the endocrine islets were more frequently found in the splenic and the third lobes, whereas they were more scarcely observed in the ventral and the dorsal lobes. This finding was also in agreement with that of previous studies in chickens,^15^ geese^10^ and young quails.^16^ Pancreatic islets show apparent varieties in distribution and location among species. Tomita *et al*. demonstrated that the highest levels of insulin, glucagon and somatostatin concentrations were found in the splenic lobe.^17^ In addition, Oakberg showed that B islets were mainly found in the splenic lobe.^18^ Weir *et al*. performed radioimmunoassay on extracts of various regions of the chicken pancreas and, in agreement with cell frequency measurements, found extremely high concentrations of glucagon and somatostatin in the splenic lobe.^19^ However, in ducks, the alpha and beta islets are reported to be numerous in all pancreatic lobes.^13^ Rawdon and Larsson are reported that the smaller beta islets are present in the dorsal and ventral lobes.^15^

The results of the present study showed that there were no alpha islets in the dorsal or ventral pancreatic lobes. This finding showed that removal of these two lobes in native chickens would probably have no effect on the glucagon production potential of the gland. In agreement with our findings, Mikami and Ono stated that the ventral lobe of the white leghorn cockerel contains no alpha islets. They stated that removal of the third and splenic lobes effectively removes the alpha cells and causes hypo-glycemia.^20^ McClish and Eglitis found relatively large dark islets in the ventral lobe of the duck pancreas and stated that removal of portions of the dorsal lobe would still leave a significant number of dark islets.^21^ However, removal of the ventral lobe and portions of the dorsal lobe near the sublobe junctions would perhaps have an effect similar to that described by Mikami and Ono for the chicken.^20^

Our results also showed that in both sexes, the mean proportion of alpha islets was significantly greater in third than in splenic lobe. Frequencies of beta islets in different lobes in male chickens were as follow: Splenic lobe > dorsal lobe > third lobe > ventral lobe and in females as: splenic lobe > dorsal lobe > ventral lobe > third lobe. No literature was available to compare these results in native chickens. Results obtained from the present study also showed that in both sexes frequencies of mixed islets in different lobes were as follow: Ventral lobe > dorsal lobe > third lobe > splenic lobe. In contrast to our observations of the native chicken pancreas, Şimşek *et al*. stated that the mean proportion of mixed islets was significantly greater in the splenic than in the dorsal and ventral lobes in falcons.^16^

The mean proportion of beta islets in the ventral and third lobes was significantly greater in male than in female chickens (*p* < 0.05). The mean proportion of mixed islets in the third and splenic lobes was significantly greater in female than in male chickens (*p* < 0.05). In addition to the genetic variations, these findings may be due the different effects of sexual steroid hormones on pancreatic structure in male and female native chickens. In addition to classical reproductive organs, many other tissues are targets of sexual steroid hormones and the pancreas which performs both endocrine and exocrine functions has proven to be an extragonadal target of sexual steroid hormone action. It has been shown that steroid hormones play an important role in susceptibility and development of diabetes in animal models.^22^ Pancreatic tissue synthesizes and transforms steroid hormones, responds to steroid hormones and expresses steroid specific receptor molecules. Some endocrine functions such as insulin synthesis and release are also modulated by steroids.^23^ Thus, the observed sexual differences in distribution of the various islets may be due to different responsiveness of the gland to male and female gonadal steroids. More researches, however, are needed for the explanation. 

This set of data creates a new paradigm for the holistic study of gender effects on avian pancreatic endocrine tissue and opens new research fields. The application of this new paradigm might result in an increase in the knowledge of pancreatic physiology, in design of new and better diagnostic methods and eventually in the design of more effective medical treatments for the pancreatic cancers.

In view of the results of the present study we can assume that the distribution of Langerhans islets in the different pancreatic lobes may be a sensitive parameter of the gonadal steroid hormones effects on the gland in male and female native chickens. 
